# Automatic Clustering of Flow Cytometry Data with Density-Based Merging

**DOI:** 10.1155/2009/686759

**Published:** 2009-11-19

**Authors:** Guenther Walther, Noah Zimmerman, Wayne Moore, David Parks, Stephen Meehan, Ilana Belitskaya, Jinhui Pan, Leonore Herzenberg

**Affiliations:** ^1^Department of Statistics, Stanford University, Stanford, CA 94305, USA; ^2^Department of Genetics, Stanford University, Stanford, CA 94305, USA

## Abstract

The ability of flow cytometry to allow fast single cell interrogation of a large number of cells has made this technology ubiquitous and indispensable in the clinical and laboratory setting. A current limit to the potential of this technology is the lack of automated tools for analyzing the resulting data. We describe methodology and software to automatically identify cell populations in flow cytometry data. Our approach advances the paradigm of manually gating sequential two-dimensional projections of the data to a procedure that automatically produces gates based on statistical theory. Our approach is nonparametric and can reproduce nonconvex subpopulations that are known to occur in flow cytometry samples, but which cannot be produced with current parametric model-based approaches. We illustrate the methodology with a sample of mouse spleen and peritoneal cavity cells.

## 1. Introduction

Flow cytometry allows to measure simultaneously multiple characteristics of thousands of cells. This ability has made flow cytometry a prevalent instrument in both the research and clinical settings. A major road block to tapping the full potential of this technology is the lack of data analysis methodology and software that allows for an automated and objective analysis of the data generated by this high-throughput instrument. One important part of the analysis of flow cytometry data is gating, that is, the identification of homogeneous subpopulations of cells. The current standard technique for this type of analysis is to draw 2D gates manually with a mouse on a computer screen, based on the user's interpretation of density contour lines that are provided by software tools such as FlowJo (http://www.treestar.com/) or BioConductor [1, 2]. The cells falling in this gate are extracted and the process is repeated for different 2D projections of the gated cells, thus resulting in a sequence of two-dimensional gates that describe subpopulations of the multivariate flow cytometry data.

There are several obvious problems with this kind of analysis. It is subjective as it is based on the user's interpretation and experience, it is error-prone, difficult to reproduce, time consuming, and does not scale to a high-throughput setting. For these reasons manual gating has become a major limiting aspect of flow cytometry [3–5], and there is a widely recognized need for more advanced analysis techniques [6, 7].

There have been several recent attempts to produce automatic and objective gates. Those employ the *k*-means algorithm [8–10] or mixture models with Gaussian components [11] or with t components and a Box-Cox transformation [12]. A drawback of all of these methods is that they produce necessarily convex subpopulations; whereas occasionally subpopulations occur that are not convex and are, for example, kidney shaped. Such subpopulations can arise, for example, when two markers are added sequentially, so that there is a developmental progression over time that moves the subpopulation first in one direction and then in another direction. The methodology introduced in this paper is grounded in nonparametric statistical theory which allows for such subpopulations.

We follow the paradigm that clusters of the data can be delineated by the contours of high-density regions [13], which is also the rationale that underlies manual gating. We implement this paradigm algorithmically by constructing a grid with associated weights that are derived by binning the data. The purpose of this grid is twofold. It allows for a fast computation of the density estimate via the Fast Fourier Transform, and it provides for an economical but flexible representation of clusters. We model each high-density region by a collection of grid points. This collection is determined algorithmically as follows. We establish links between certain neighboring grid points based on statistical decisions regarding the gradient of the density estimate. The goal is to connect neighboring grid points by a chain of links that follow the density surface “uphill.” The result of this first processing stage is a number of chains that link certain grid points and which either terminate at the mode of a cluster or represent background that will not be assigned to a cluster. In a second stage the algorithm will combine some of these chains if statistical procedures indicate that they represent the same cluster. The idea of following the gradient uphill to determine clusters is motivated by manual gating and is similar to a proposal by [14], which albeit does not provide the statistical methodology required to make decisions about nonzero gradients and combining certain chains. Reference [15] gives a visual display of gradients but no algorithm for finding clusters by linking the gradients.

The end result of our algorithm is clusters that are represented by chains that link certain grid points. This representation has the advantage that it provides an efficient data structure for visualizing and extracting the cells that belong to a cluster. The chains that link grid points in a cluster represent a tree structure which can be traversed backwards to efficiently enumerate all grid points in the cluster and hence to retrieve all cells in the cluster via their nearest neighbor grid point.

## 2. Methods

### 2.1. Representing the Distribution on a Grid

Binning data on a grid allows fast processing with little loss of accuracy [16]. The current software implementation of our methodology works with successive 2D projections and we describe the methodology in this setting, although the algorithm can be generalized to work in higher dimensions from the start.

Thus we have *n* data points *x*_*i*_ = (*x*_*i*1_, *x*_*i*2_), *i* = 1,…, *n*. To construct a grid we choose a positive integer *M*, typically *M* = 128 or 256, and construct the grid consisting of *M* ^2^ points as follows. Set Δ_*j*_ = (max _*i*_ *x*_*i*,*j*_ − min _*i*_ *x*_*i*,*j*_)/(*M* − 1), *j* = 1, 2, and define the *j*th coordinate of *y*_(*m*_1_,*m*_2_)_ to be *y*_*m*_*j*__ = min _*i*_ *x*_*i*,*j*_ + (*m*_*j*_ −1)Δ_*j*_, *m*_*j*_ = 1,…, *M*. Then the grid is defined as {*y*_(*m*_1_,*m*_2_)_ : (*m*_1_, *m*_2_) ∈ {1,…, *M*}^2^}.

Next, each grid point *y*_**m**_, where **m** = (*m*_1_, *m*_2_) ∈ {1,…, *M*}^2^, is assigned a weight *w*_**m**_ by linearly binning [16] the observations *x*_*i*_, that is,

(1) $$\begin{array}{c} w_{m}=\overset{n}{\underset{i=1}{\sum }}\overset{2}{\underset{j=1}{\prod }}\max\;\left(0,1-\frac{\left|x_{i,j}-y_{m_{j}}\right|}{\Delta_{j}}\right). \end{array}$$

The grid {*y*_**m**_, **m** ∈ {1,…, *M*}^2^} and the associated weights {*w*_**m**_, **m** ∈ {1,…, *M*}^2^} represent an approximation to the cell distribution. Our software implementation allows the user to choose various values of *M*. A larger choice of *M* results in a finer grid and hence a more precise approximation of the cell distribution at the expense of more computing time. However, in accordance with the results in [16], we found that a relatively small number of bins already give an excellent approximation. Within a precision of 0.01% of the total cell population we could not detect a change in the outcome of gating small subpopulations when increasing *M* from our default value of 256 to 512.

Our clustering algorithm described below uses only the grid and the associated weights to derive the clustering assignment. This assignment is then applied to cluster observations *x*_*i*_ as follows. Each observation *x*_*i*_ is assigned to the grid point *y*_**m**_ that is the closest to *x*_*i*_ in Euclidean norm. Then *x*_*i*_ is assigned to the same cluster to which its associated grid point *y*_**m**_ is assigned. Likewise, all observations assigned to a certain cluster can be retrieved as follows. Find all grid points *y*_**m**_ assigned to the given cluster, then find all observations *x*_*i*_ that are assigned to these grid points.

### 2.2. Computing the Estimate of the Cell Density

At each grid point *y*_**m**_, **m** ∈ {1,…, *M*}^2^, an estimate of the density surface $\hat{f}(y_{m})$ is computed as follows.

Denote by $\phi (x)=1/\sqrt{2\pi }\exp\;(-x^{2}/2)$ the Gaussian kernel. Then the estimated density at *y*_**m**_ is given by (see, e.g., [16])

(2) $$\begin{array}{cc} \hat{f}\left(y_{m}\right) & =\frac{1}{n}\overset{Z_{1}}{\underset{l_{1}=-Z_{1}}{\sum }}\overset{Z_{2}}{\underset{l_{2}=-Z_{2}}{\sum }}w_{m-l}\times \overset{2}{\underset{j=1}{\prod }}\frac{\phi \left(l_{j}\Delta_{j}/h_{j}\right)}{h_{j}}, \end{array}$$

where **l** = (*l*_1_, *l*_2_), *Z*_*j*_ = min (⌊4*h*_*j*_/Δ_*j*_⌋, *M* − 1), and *h*_*j*_ = SD({*x*_*i*,*j*_, *i* = 1,…, *n*})*n* ^−1/6^, where SD denotes standard deviation. The above sum can be computed quickly with the Fast Fourier Transform (FFT) in a well-known way [16], but it can also be computed directly using the above formula without the FFT.

### 2.3. Association Pointers between the Grid Points

First, for each grid point we compute the standard error of the corresponding density estimate and then label those grid points as background whose density does not pass a certain statistical threshold. The interpretation of this criterion is that it tests whether the density is significantly different from zero; see Step 1 for details.

Next we want to construct links between grid points that follow the density gradient, that is, point “uphill.” To this end, we visit each grid point in turn and compare the density estimate on this grid point with those of its neighboring grid points, of which there are at most eight. We establish a link to that neighboring grid point that has the highest value of the density estimate, provided that the difference in density estimates is statistically significant (Step 2). Testing whether the latter difference is nonzero is necessary as otherwise the variability of the density estimate may lead to links that may accidentally connect different clusters. Computationally we implement links by way of the programming language data type of a pointer.

Next we follow each chain to its end and determine whether it represents a cluster or background (Step 3). Then we determine whether two clusters need to be merged because they are connected by a path that possesses no statistically significant trough (Step 4). This is done by iteratively building a set of grid points which are neighbors to a local maximum of the density surface, are not maxima or background, and do not exhibit a statistically significant change in density when compared to the local maximum. If this set in turn possesses a neighboring grid point that is a local maximum, then we found a path (via this set) between two local maxima that does not exhibit a statistically significant trough. Consequently the last part of Step 4 merges the corresponding clusters by establishing pointers to the grid point with the highest density. We iterate Step 4 until there are no more changes in the clusters (Step 5). It can be shown that there will be only finitely many iterations. Step 6 takes care of remaining points that are assigned to the background. Thus the resulting number of clusters is determined by the data via the statistical methodology described previously.

Here is a more formal description of the various steps.

**Step 1**

Consider all grid points *y*_**m**_, **m** ∈ {1,…, *M*}^2^, in turn. For each grid point *y*_**m**_ compute

(3) $$\begin{array}{cc} {\hat{\sigma }}_{m}^{2} & =\frac{1}{n\left(n-1\right)}\overset{Z_{1}}{\underset{l_{1}=-Z_{1}}{\sum }}\overset{Z_{2}}{\underset{l_{2}=-Z_{2}}{\sum }}w_{m-l}\\ & \;\times \overset{2}{\underset{j=1}{\prod }}\frac{\phi^{2}\left(l_{j}\Delta_{j}/h_{j}\right)}{h_{j}^{2}}-\frac{1}{n-1}\hat{f}{\left(y_{m}\right)}^{2}. \end{array}$$

${\hat{\sigma }}_{m}$ is an estimate of the standard error of the estimated density at *y*_**m**_. ${\hat{\sigma }}_{m}^{2}$ can be computed with the FFT as above. Define the index set $𝒮=\{m\in \{1,\ldots ,M{\}}^{2}:\hat{f}(y_{m})>4.3*\sqrt{{\hat{\sigma }}_{m}^{2}}\}$. The factor 4.3 is an adjustment for multiple testing over the grid and is obtained by calculations as in [15]. Thus *𝒮* is the set of grid points, where the density is significantly different from zero. Grid points outside this set are marked as background. From each grid point *y*_**m**_, **m** ∉ *𝒮*, a pointer is established that points to a dummy state that represents background noise.

**Step 2**

For all grid points *y*_**m**_, **m** ∈ *𝒮*, in turn.

Consider all the neighboring grid points *p*_1_,…, *p*_*n*_*m*__, which are defined as the set of all grid points contained in the box ⋂_*j* = 1_^2^{*x* : *y*_*m*_*j*__ − Δ_*j*_ ≤ *x*_*j*_ ≤ *y*_*m*_*j*__ + Δ_*j*_}. Let *p* ∈ {*p*_1_,…, *p*_*n*_*m*__} such that $\hat{f}(p)={\max\;}_{k=1,\ldots ,n_{m}}\hat{f}(p_{k})$, splitting ties in an arbitrary manner. Then establish an association pointer from *y*_**m**_ to *p* provided the following two conditions hold:

$\hat{f}(p)>\hat{f}(y_{m})$ and $\left(\partial /\partial e\right)\hat{f}(y_{m})>\lambda_{m}$, where *e* = (*p* − *y*_**m**_)/||*p* − *y*_**m**_||, || · || denotes Euclidean norm, and $\left(\partial /\partial e\right)\hat{f}(y_{m})$ and *λ*_**m**_ are defined as follows:

(4) $$\begin{array}{cc} & \frac{\partial }{\partial e}\hat{f}\left(y_{m}\right)=\overset{2}{\underset{a=1}{\sum }}e_{a}\frac{\partial }{\partial y_{m_{a}}}\hat{f}\left(y_{m}\right),\\ & \frac{\partial }{\partial y_{m_{a}}}\hat{f}\left(y_{m}\right)=\frac{1}{n}\overset{Z_{1}}{\underset{l_{1}=-Z_{1}}{\sum }}\overset{Z_{2}}{\underset{l_{2}=-Z_{2}}{\sum }}w_{m-l}\times \frac{-l_{a}\Delta_{a}}{h_{a}^{2}}\overset{2}{\underset{j=1}{\prod }}\frac{\phi^{}\left(l_{j}\Delta_{j}/h_{j}\right)}{h_{j}^{}},\\ & \lambda_{m}=q\left(0.95^{1/\kappa }\right)\sqrt{{\hat{\Sigma }}_{m}^{2}},\\ & \kappa =\frac{\#𝒮\sum_{m\in 𝒮}^{}w_{m}}{n2\pi \prod_{j=1}^{2}h_{j}\sum_{m\in 𝒮}^{}\hat{f}\left(y_{m}\right)},\\ & {\hat{\Sigma }}_{m}^{2}=\frac{1}{n-1}\left(\overset{2}{\underset{a,b=1}{\sum }}e_{a}e_{b}\left[A-\frac{\partial }{\partial y_{m_{a}}}\hat{f}\left(y_{m}\right)\frac{\partial }{\partial y_{m_{b}}}\hat{f}\left(y_{m}\right)\right]\right),\\ & A=\frac{1}{n}\overset{Z_{1}}{\underset{l_{1}=-Z_{1}}{\sum }}\overset{Z_{2}}{\underset{l_{2}=-Z_{2}}{\sum }}w_{m-l}\times \frac{l_{a}l_{b}\Delta_{a}\Delta_{b}}{h_{a}^{2}h_{b}^{2}}\overset{2}{\underset{j=1}{\prod }}\frac{\phi^{2}\left(l_{j}\Delta_{j}/h_{j}\right)}{h_{j}^{2}}. \end{array}$$

Here *e*_1_, *e*_2_ denote the standard Euclidean basis vectors. ${\hat{\Sigma }}_{m}^{2}$ is an estimate of the variance of $\left(\partial /\partial e\right)\hat{f}(y_{m})$ and *q*(0.95^1/*κ*^) is the normal distribution critical value adjusted for multiple testing via *κ*; see, for example, [15]. *A* is an estimate of (∂/∂*y*_*m*_*a*__)*f*(*y*_**m**_)(∂/∂*y*_*m*_*b*__)*f*(*y*_**m**_). *q*(*x*) denotes the 100 · *x*th percentile of the standard normal distribution. All the sums can be computed with the FFT as above. Checking that the derivative at *y*_**m**_ in the direction of *p* is significant, rather than just linking *y*_**m**_ to *p*, prevents an accidental linking of different clusters. However, this approach may result in not being able to establish links near the maximum, where the density surface is flat. This is addressed by Step 4, which merges such grid points.

**Step 3**

For all grid points *y*_**m**_, **m** ∈ *𝒮*, in turn.

If a pointer originates at *y*_**m**_, then it will point to a different grid point, which itself may have a pointer originating from it. This succession of pointers is followed until one arrives at a grid point *y*_**z**_ that either

- *y*_**z**_ does not have any pointer originating from it, or
- *y*_**z**_ has a pointer originating from it which points to a dummy state that represents a cluster or background noise.

In case (a) all the pointers visited in succession will be removed and new pointers originating from each grid point visited in succession will be established to the dummy state that represents the background noise, provided the following condition holds:

(5) $$\begin{array}{c} \hat{f}\left(y_{z}\right)<q\left(0.95^{1/\kappa }\right)\sqrt{{\hat{\sigma }}_{z}^{2}}. \end{array}$$

Otherwise, provided there is a pointer into *y*_**z**_, then a new pointer will be established that originates from *y*_**z**_ and points to a newly established dummy state that represents a new cluster.

In case (b) no pointers are removed or established.

**Step 4**

Let {*y*_**m**(1)_, …*y*_**m**(*k*)_} be the set of all grid points which have a pointer originating from them to a dummy state representing a cluster, enumerated such that $\hat{f}\left(y_{m\left(1\right)}\right)\geq \cdots \geq \hat{f}(y_{m(k)})$.

For *i* = 1,…, *k* do the following.

Set *𝒜* = {**m**(*i*)}. Iterate the following loop until no more indices are added to *𝒜*:

(Begin loop)

For each index **a** ∈ *𝒜* in turn, add all the indices **p** to *𝒜* that satisfy

- *y*_**p**_ is a neighbor of *y*_**a**_ as defined in Step 2,
- no pointer originates from *y*_**p**_,
- $\hat{f}(y_{p})+{\hat{\sigma }}_{p}\geq \hat{f}(y_{m(i)})-{\hat{\sigma }}_{m(i)}$

(End loop)

Denote by *ℬ* the set of indices of grid points which satisfy the following two conditions. The grid point possesses a pointer originating to a dummy state representing a cluster, and the grid point has some *y*_**p**_, **p** ∈ *𝒜* as neighbor. If *ℬ* is not empty, then do the following.

Define **q** by $\hat{f}(y_{q})={\max\;}_{r\in ℬ}\hat{f}(y_{r})$, breaking ties arbitrarily.

Establish a pointer from each *y*_**p**_, **p** ∈ *𝒜* \ {**m**(*i*)}, to *y*_**q**_.

For each **r** ∈ *ℬ*, **r** ≠ **q**, remove the pointer from *y*_**r**_ to the dummy state representing a cluster and establish a new pointer from *y*_**r**_ to *y*_**q**_.

(End loop over *i*)

**Step 5**

Repeat Step 4 until there are no more additions or deletions of pointers to dummy states representing clusters.

**Step 6**

From each grid point that does not have a pointer originating from it, establish a pointer pointing to the dummy state that represents the background noise.

After Step 6 every grid point has a pointer originating from it. Following the succession of pointers leads to a dummy state which represents either background noise or a cluster. All grid points which are thus linked to the same dummy state pertain to the same cluster (or background noise). Cluster memberships of observations *x*_*i*_ derive from the cluster memberships of the grid points as explained in Section 2.1.

## 3. Results

We implemented the density-based merging (DBM) algorithm in a Java application with a graphical user interface that allows cluster visualization and sequential selection of clusters to support progressive gating. To enable comparison of DBM gating with data gated manually with a commercial analysis package (FlowJo, http://www.treestar.com/), we record cluster assignments for each event in association with the original data. These values are used as synthetic gating parameters in the commercial package, where we can directly compare results.

Mouse spleen and peritoneal cavity cells harvested in serum-containing medium were incubated on ice for 15 minutes with a 10-color staining combination. Data were collected on an LSR II (Becton Dickinson).****

**Figure 1:**
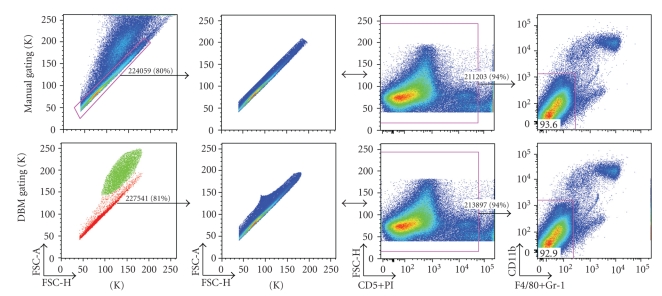
Comparison of manual and DBM gating in the scatter dimensions—singlet gates are shown as determined by the researcher (top) and DBM (bottom, colored plot frames) for neonatal mouse spleen cells. The subset is further gated using the researcher's live/dead gate and displayed in context of the next gating decision by the researcher. Note that the results of the DBM clustering are displayed with same software that was used for the manual gating (FlowJo). This was done to facilitate the comparison and because a suitable display system for publication has not yet been developed. Thus in the bottom left plot, color is used to code the clusters found by DBM.

**Figure 2:**
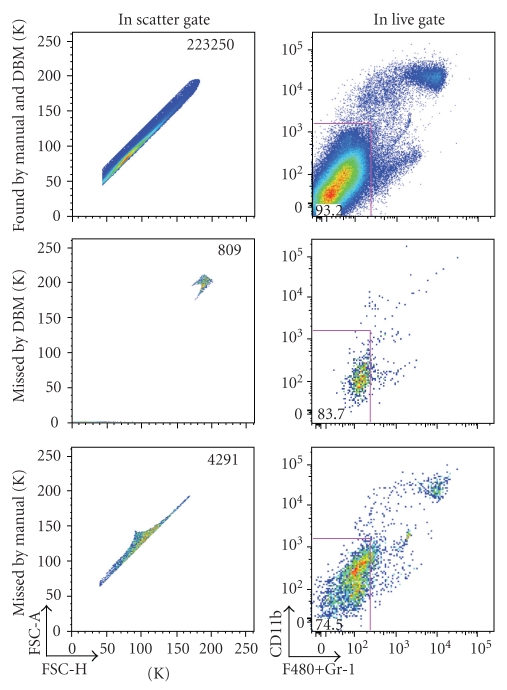
Differences in manual versus DBM gating in scatter dimensions—cells included by both gates (top), cells included in the manual gate and excluded by the DBM gate (middle), and cells included in the DBM gate and excluded by the manual gate (bottom) are displayed (column 1). Cells are live/dead gated as described in the text, and shown in the context of the next manual gating decision (column 2).

**Figure 3:**
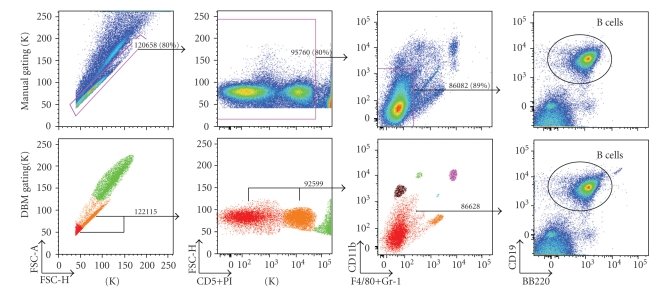
Comparison of manual and DBM gating for 3-step gating sequence—adult mouse spleen cells are analyzed using the researcher's manual gates (top plots) and the corresponding clusters identified by DBM (bottom plots with colored plot frames). Color is used to code the clusters found by DBM in the first three plots on bottom. Each of the manual/DBM gate pairs has <4% difference in total number of cells. In this study, the researcher is interested in B cells (column 4).

In the data shown in Figures 1–3, we replicate manual gating decisions from a dataset previously analyzed by a senior researcher using FlowJo. The researcher has sequentially selected gates that progressively restrict the inclusion of cells to ultimately encompass a known functionally distinct subset. For each of these sequential manual gating decisions, we select the corresponding cluster(s) defined by the DBM algorithm. In our analysis, we thus reproduce the existing workflow of the researcher, with the notable exception that we use gating boundaries that are defined algorithmically.

Figure 1 (first column) compares the initial gating in the forward-scatter area/height dimensions performed manually (top) or with DBM (bottom). The research intention here is to separate single cells from doublets and other debris. Drawing the manual gate requires a great deal of experience for a researcher to draw, owing to the lack of visual differentiation between the overlapping populations. DBM identifies two clusters that agree surprisingly well with the manual gate: the red cluster contains 81% of the total events; the corresponding expert gate contains 80% of the total events; the overlap between the two gates is 98%.

Two views of the events encompassed by the clusters are shown in columns 2 and 3 of Figure 1. Column 4 shows further gating of the samples with the same manual gate applied to the manually gated (top) and DBM gated (bottom) data shown in columns 2 and 3. The similarity of the yield from the manually gated and DBM gated sample underscores the strong overlap between the two samples.

In each case, a small percentage of the events captured by one of the gating methods are excluded from the other (Figure 2). Importantly we find that the DBM gate tends to better capture the desired events then does the researcher's gate. We define desirable events as those included in the subsequent gates that the expert set. The gate set by the expert included fewer cells in the desired subset than the DBM gate, resulting in a loss of desired cells (3474 cells). The expert gate also included fewer cells outside the desired subset. However, the additional “nondesired” cells included in the DBM gate are not relevant since the expert has gated these out of the subsequent analysis. Thus, in this situation, the DBM gate is more successful than the expert gate.

In Figures 1 and 2, we analyzed the results of a single DBM gate generated to match the first gate that the expert applied in the gating series. Figure 3, which is based on a different dataset, compares results from three sequential gates applied by the researcher with the comparable sequential DBM gates. The researcher has chosen three sequential gates (Figure 3, top): the first gate excludes doublets and debris; the second gate excludes dead cells (bright PI); the third, which yields a subset that is enriched for B cells (the target of interest to the expert), excludes monocytes and macrophages (CD11bbr, F4/80+GR-1br).

Applying the corresponding sequence of DBM clusters results in a distribution (Figure 3, bottom) that is almost indistinguishable from the distribution obtained with the expert's gates. The principal differences is a small increase in the number of cells in the B cell subset desired by the expert, and the inclusion of a small percentage of cells that lie near, but not within, the B cell subset.

We view these results as extremely promising. We are pleased that the DBM algorithm performed at least as well than the expert in terms of identifying the subset of interest in this study. We plan to perform future studies with more diverse datasets to provide a more detailed investigation of the performance of the DBM algorithm.

## 4. Discussion

Flow cytometry allows to separate cells into subsets for further analysis. The potential of this technology is currently limited by a lack of automatic and objective data analysis and gating techniques. We introduced methodology and demonstrated a software implementation that allows automatic 2D gating that is based on statistical theory and hence objective, reproducible, and fast. Typically, the automatic gating takes only a fraction of a second. An important feature of this methodology is that it is nonparametric and allows for nonconvex gates, which current parametric methodology with mixture models does not provide. Likewise, the nonparametric statistical theory provides the information necessary to decide on the number of populations in the sample, which is known to be a difficult problem in the context of parametric mixture models with no satisfactory solution currently available.

We implemented our methodology in a sequential 2D setting to automate the traditional manual gating. While the methodology can in principle be implemented in a higher-dimensional setting, there are also advantages to stick with the traditional sequential procedure. First, many users are familiar with the sequential gating procedure and may be hesitant to work with the high-dimensional output of a “black box,” which may be difficult to interpret. Second, it is common practice to first project the data on the forward light scatter (FSC) and sideward light scatter (SSC) to distinguish basic cell types (e.g., monocytes and lymphocytes) and to remove dead cells and cell debris. Also, the user may have prior knowledge that leads her to consider certain 2D projections or gating paths. These aspects are readily incorporated in our implementation. Third, sequential 2D gating allows for an informative and straightforward visualization of the gating and the results.

We implemented our methodology in software called ClusterGenie which we plan to be open source but distributed commercially. We demonstrated it on a sample of mouse spleen and peritoneal cavity cells. Our results compared favorably with expert gating of the data in FlowJo. We plan a rigorous quantitative assessment of our methodology in the near future.
